# Predictive models and biomarkers for survival in stage III breast cancer: a review of clinical applications and future directions

**DOI:** 10.1097/MS9.0000000000002517

**Published:** 2024-08-30

**Authors:** Emmanuel Ifeanyi Obeagu, Getrude Uzoma Obeagu

**Affiliations:** a Department of Medical Laboratory Science, Kampala International University; b School of Nursing Science, Kampala International University, Ishaka, Uganda

**Keywords:** biomarkers, breast cancer, cancer, clinical parameters, genetic markers

## Abstract

Stage III breast cancer, characterized by locally advanced tumors and potential regional lymph node involvement, presents a formidable challenge to both patients and healthcare professionals. Accurate prediction of survival outcomes is crucial for guiding treatment decisions and optimizing patient care. This publication explores the potential clinical utility of predictive tools, encompassing genetic markers, imaging techniques, and clinical parameters, to improve survival outcome predictions in stage III breast cancer. Multimodal approaches, integrating these tools, hold the promise of delivering more precise and personalized predictions. Despite the inherent challenges, such as data standardization and genetic heterogeneity, the future offers opportunities for refinement, driven by precision medicine, artificial intelligence, and global collaboration. The goal is to empower healthcare providers to make informed treatment decisions, ultimately leading to improved survival outcomes and a brighter horizon for individuals facing this challenging disease.

## Introduction

Highlights

- Significance: predictive models and biomarkers are crucial for personalizing treatment in stage III breast cancer.
- Current applications: these tools help identify high-risk patients and tailor therapeutic strategies.
- Biomarkers: common biomarkers include ER, PR, HER2, and Ki-67.
- Emerging technologies: genomic and proteomic profiling are advancing predictive accuracy.
- Future directions: integrating AI and machine learning could enhance predictive models for better outcomes.

Stage III breast cancer is an advanced stage of the disease characterized by extensive regional lymph node involvement and possible local tissue invasion^1^. At this stage, the cancer has spread beyond the primary tumor site into nearby lymph nodes but has not yet metastasized to distant organs. This stage is significant because it presents a higher risk of recurrence and poorer prognosis compared to earlier stages of breast cancer. Accurate prediction of survival outcomes at this stage is crucial for devising effective treatment strategies and improving patient outcomes^2,3^. Predicting survival outcomes for Stage III breast cancer patients is fundamental for personalizing treatment plans and managing the disease effectively^4^. Traditional approaches to treatment are often guided by clinical staging, pathological features, and historical treatment outcomes. However, with the increasing complexity of breast cancer biology, there is a growing need for advanced predictive models and biomarkers that provide more precise and individualized survival predictions. These tools help clinicians evaluate the likelihood of disease recurrence, choose appropriate therapies, and monitor patient responses, ultimately aiming to improve survival rates and quality of life^5,6^. Historically, predictive modeling for breast cancer has relied on clinical and pathological factors, such as tumor size, lymph node involvement, and histological grade^7^. The TNM staging system, developed by the American Joint Committee on Cancer (AJCC), and the Nottingham Prognostic Index (NPI) have been foundational in predicting patient outcomes^8^. While these models have been instrumental in guiding treatment decisions, they have limitations related to their reliance on static, one-dimensional data and their inability to incorporate new molecular and genetic information that may affect prognosis.

Recent advancements in technology have significantly enhanced the ability to predict survival outcomes for Stage III breast cancer^9^. Machine learning and artificial intelligence (AI) technologies have introduced sophisticated analytical methods that can process large and complex datasets to uncover patterns and make predictions. These technologies offer the potential for more accurate and dynamic predictive models by integrating diverse types of data, including genetic, transcriptomic, and clinical information. Biomarkers have become critical tools in predicting survival outcomes for Stage III breast cancer. Established biomarkers such as HER2, estrogen receptor (ER), and progesterone receptor (PR) status provide valuable insights into tumor biology and response to therapy. Additionally, newer biomarkers and genomic assays, including Oncotype DX and MammaPrint, offer advanced methods for assessing the risk of recurrence and guiding treatment decisions. Machine learning and AI have revolutionized predictive modeling in oncology by offering advanced algorithms and models that improve the accuracy of survival predictions^10^. Techniques such as logistic regression, support vector machines, and neural networks have been employed to analyze large datasets and identify prognostic factors. These approaches allow for the development of more sophisticated models that can predict patient outcomes with greater precision. The integration of multiomics data, including genomic, proteomic, and transcriptomic information, represents a significant advancement in predictive modeling for Stage III breast cancer^11^. Multiomics approaches enable the creation of comprehensive models that consider various aspects of tumor biology and patient health. By combining different types of biological data, researchers can develop more accurate and individualized predictive tools. This section explores the benefits and challenges of integrating multiomics data into predictive models for breast cancer survival. Predictive models and biomarkers have practical applications in clinical settings for guiding treatment decisions, monitoring disease progression, and evaluating therapeutic responses. These tools help clinicians stratify patients based on their risk of recurrence and select appropriate therapies^12^.

## Aim

The aim of this review is to comprehensively evaluate and synthesize the current knowledge on predictive models and biomarkers for assessing survival outcomes in Stage III breast cancer.

## Rationale

The rationale for this review stems from the critical role that predictive models and biomarkers play in managing Stage III breast cancer, a complex and heterogeneous disease. Stage III breast cancer, characterized by local and regional disease progression, presents significant challenges in treatment and prognosis due to its advanced nature and diverse patient profiles. Accurate predictive models and reliable biomarkers are fundamental for assessing disease progression, selecting appropriate therapies, and forecasting survival outcomes. Despite advancements in these areas, there remains a need for more refined and effective tools to guide clinical decision-making and enhance treatment personalization. Recent developments in biomarker research have unveiled new opportunities for improving breast cancer management. Hormone receptor status, HER2 expression, and Ki-67 proliferation indices are well-established biomarkers that guide current treatment protocols. However, there is a growing body of evidence suggesting that newer biomarkers and advanced genomic technologies could further refine prognostic predictions and treatment strategies. This review addresses these advancements and evaluates their potential clinical impact. Predictive models based on clinical, genomic, and molecular data offer a sophisticated approach to forecasting patient outcomes. The integration of these models into clinical practice could revolutionize how Stage III breast cancer is managed by providing tailored treatment plans based on individual patient profiles. The review explores existing models, assesses their effectiveness, and discusses how these models can be improved and incorporated into standard practice. As the field of breast cancer research continues to evolve, there is a need to explore future directions that could lead to breakthroughs in predictive modeling and biomarker discovery. By identifying emerging technologies and research trends, this review aims to guide future investigations and encourage innovation in the quest for better survival predictions and personalized treatment options for Stage III breast cancer.

## Review methodology

### Literature search strategy

To ensure a thorough and systematic review, a comprehensive literature search was conducted using multiple academic databases. These databases included:

1. PubMed: a premier resource for biomedical literature.
2. Google Scholar: for accessing a broad range of academic articles and citations.
3. Web of Science: to find high-quality research articles and reviews.
4. Scopus: for its extensive coverage of peer-reviewed journals and conference proceedings.

The search strategy involved using a combination of keywords and Boolean operators to capture relevant studies. The primary search terms included ‘Stage III Breast Cancer’, ‘Predictive Models’, ‘Biomarkers’, ‘Survival Outcomes’, ‘Clinical Applications’, and ‘Future Directions’. These keywords were used both individually and in combination to identify articles that addressed predictive models, biomarkers, and their clinical implications for survival in Stage III breast cancer. The search was limited to studies published in the last two decades to ensure the relevance and currency of the information.

### Selection criteria

The selection of articles was guided by specific inclusion and exclusion criteria to ensure that the review focused on high-quality and relevant studies. The inclusion criteria were as follows:

1. Study type: peer-reviewed research articles, systematic reviews, meta-analyses, and clinical guidelines.
2. Publication date: studies published from 2003 to 2023 to ensure that the review reflects recent advancements and developments.
3. Focus: articles addressing predictive models and biomarkers for survival outcomes in Stage III breast cancer.
4. Language: publications in English to ensure comprehension and consistency in analysis.

Exclusion criteria included studies that were not related to Stage III breast cancer, those focusing on preclinical research, and articles that did not provide relevant data on predictive models or biomarkers. Studies that did not meet the methodological quality standards or were of insufficient detail were also excluded.

### Data extraction and analysis

Data extraction was performed systematically by reviewing the selected articles to extract relevant information. This process involved:

1. Identifying key variables: extracting data on the types of biomarkers studied (e.g. ER, PR, HER2, and Ki-67), predictive models used, their clinical applications, and their effectiveness in predicting survival outcomes.
2. Summarizing findings: organizing information on the clinical relevance of biomarkers and models, their applications in practice, and the results of recent studies.
3. Evaluating study quality: assessing the quality of the studies based on criteria such as study design, sample size, methodology, and relevance to Stage III breast cancer.

The extracted data was then analyzed to identify common themes, trends, and gaps in the current research landscape. The analysis focused on evaluating the effectiveness of predictive models, the clinical utility of biomarkers, and the implications for future research and clinical practice.

### Synthesis of findings

The synthesis of findings involved grouping the data into thematic categories, including:

1. Clinical applications of biomarkers: how existing biomarkers are used in clinical practice to guide treatment decisions and predict patient outcomes.
2. Predictive models for survival outcomes: review of various predictive models, including statistical and machine learning approaches, and their effectiveness in estimating survival outcomes for Stage III breast cancer patients.
3. Emerging technologies and future directions: exploration of new biomarkers, genomic technologies, and future research opportunities in the field of breast cancer management.

### Genetic markers of stage III breast cancer

Genetic markers play a critical role in understanding and predicting the behavior of stage III breast cancer. These markers provide insights into the underlying molecular characteristics of the tumor, which can help healthcare professionals make more informed treatment decisions and estimate a patient’s prognosis. Here are some key genetic markers that are commonly associated with stage III breast cancer:

1. Estrogen Receptor (ER), Progesterone Receptor (PR), and Human Epidermal Growth Factor Receptor 2 (HER2): these receptors are essential biomarkers in breast cancer. Estrogen and progesterone receptors are hormone receptors, and their presence indicates that the cancer is hormone receptor-positive. HER2 is a protein involved in cell growth, and HER2-positive breast cancer is associated with more aggressive tumors^13^. The status of these receptors helps determine treatment options, such as hormone therapy and targeted HER2 therapies.
2. Ki-67 (Proliferation Marker): Ki-67 is a protein that measures the rate of cell division in a tumor. High Ki-67 levels are associated with more aggressive breast cancers^14^. It can help healthcare professionals assess the tumor’s growth rate and guide treatment decisions.
3. BRCA1 and BRCA2 mutations: mutations in the BRCA1 and BRCA2 genes are associated with an increased risk of developing breast cancer^15^. In stage III breast cancer, identifying these mutations can have implications for treatment and risk assessment. Patients with BRCA mutations may be candidates for targeted therapies, and their family members may benefit from genetic counseling and testing.
4. TP53 mutations: TP53 is a tumor suppressor gene, and mutations in this gene are linked to various cancers, including breast cancer^16^. TP53 mutations can influence the aggressiveness of the tumor and the response to treatment. Identifying TP53 mutations may guide therapy decisions and prognosis assessment.
5. PIK3CA mutations: The PIK3CA gene encodes a protein involved in cell growth and signaling. Mutations in PIK3CA are common in breast cancer and can affect the response to certain treatments, such as PI3K inhibitors^17^. Identifying PIK3CA mutations may help select targeted therapies.
6. Gene expression profiling: tests like Oncotype DX, Mammaprint, and Prosigna analyze the expression of multiple genes in breast cancer tissue^18^. These tests provide a score that predicts the risk of recurrence and can help determine the need for adjuvant chemotherapy.
7. MicroRNA profiles: MicroRNAs are small RNA molecules that regulate gene expression. Altered microRNA profiles have been associated with breast cancer progression and may serve as potential markers for predicting survival outcomes^19^.
8. Tumor mutational burden (TMB): TMB measures the number of genetic mutations in a tumor. High TMB can indicate a more aggressive tumor and may influence treatment choices, such as immunotherapy^20^.

### Imaging techniques in stage III breast cancer

Imaging techniques play a crucial role in the diagnosis, staging, and management of stage III breast cancer. These techniques help healthcare professionals assess the extent of disease, determine the best treatment approach, and monitor the response to therapy. Here are some of the key imaging techniques used in the evaluation of stage III breast cancer:

1. Mammography: mammography is the most common imaging tool for breast cancer screening and diagnosis. In stage III breast cancer, mammography can reveal the presence of a tumor and provide information about the size, location, and characteristics of the cancer^21^. It is especially useful for identifying microcalcifications, which can be indicative of cancer.
2. Ultrasound: breast ultrasound is often used as a complementary imaging technique to mammography^22^. It is particularly helpful in distinguishing between solid and cystic lesions and can provide valuable information about the tumor’s size, shape, and vascularity. Ultrasound can also guide needle biopsies.
3. Breast MRI: Breast MRI is a highly sensitive imaging technique that can provide detailed information about the extent of disease in stage III breast cancer^23^. It is particularly useful for evaluating the presence of multifocal or multicentric disease, assessing tumor size, and determining the involvement of lymph nodes. Dynamic contrast-enhanced MRI (DCE-MRI) is commonly used to visualize blood flow within the breast tissue, which can aid in the diagnosis and staging of breast cancer.
4. Positron emission tomography (PET) Scan: PET scans with fluorodeoxyglucose (FDG-PET) can help assess the metabolic activity of breast cancer cells^24^. FDG-PET scans are valuable in determining the stage of breast cancer, particularly by detecting the spread of cancer to distant sites (metastasis). This information can impact treatment decisions.
5. Computed tomography (CT) scan: CT scans are used to evaluate the chest, abdomen, and pelvis for the presence of distant metastases in stage III breast cancer^25^. It can help assess the involvement of nearby lymph nodes, organs, or bones.
6. Chest X-ray: A chest X-ray may be performed to assess the presence of lung metastases. It is a quick and noninvasive way to identify potential spread to the lungs.
7. Sentinel lymph node biopsy (SLNB): While not strictly an imaging technique, SLNB is a procedure that uses a radiotracer and/or blue dye to identify and biopsy the sentinel lymph node(s) closest to the primary breast tumor^26^. This procedure helps determine whether cancer has spread to the lymph nodes.
8. 3D mammography (Tomosynthesis): this advanced form of mammography provides three-dimensional images of the breast, enhancing the ability to detect and characterize breast lesions. It may be used in addition to standard mammography to improve diagnostic accuracy.
9. Contrast-enhanced mammography: This newer imaging technique combines mammography with intravenous contrast to highlight blood flow and enhance the visualization of breast lesions. It can be particularly useful in evaluating the vascularity of tumors.

### Predictive models for survival outcomes in stage III breast cancer

Predictive models are essential tools in oncology for estimating survival outcomes and guiding treatment decisions for Stage III breast cancer patients^27^. These models integrate various prognostic factors to predict disease progression, recurrence risk, and overall survival. Traditional predictive models rely on clinical and pathological data, while recent advancements incorporate advanced statistical methods and machine learning techniques to enhance prediction accuracy. This section explores different types of predictive models used for assessing survival outcomes in Stage III breast cancer and their applications in clinical practice. Traditional predictive models for Stage III breast cancer primarily use clinical and pathological parameters to estimate survival outcomes^28^. These models include the TNM Staging System, which evaluates tumor size (T), regional lymph node involvement (N), and distant metastasis (M) to stage the disease and predict prognosis. Another widely used model is the Nottingham Prognostic Index (NPI), which combines tumor size, grade, and lymph node status to predict patient outcomes. While these models provide a foundation for understanding prognosis, they have limitations in capturing the full complexity of breast cancer biology and predicting individual patient outcomes.

Statistical models and risk scores offer more refined approaches for predicting survival outcomes. The Adjuvant Online tool and the PREDICT model are two prominent examples that use statistical algorithms to estimate the probability of disease recurrence and survival^29^. These models incorporate factors such as age, tumor grade, hormone receptor status, and HER2 status to calculate risk scores and provide personalized treatment recommendations. Despite their utility, these models can be limited by their reliance on historical data and the static nature of their input variables. Recent advancements in machine learning have introduced new methodologies for predicting survival outcomes in Stage III breast cancer^30^. Techniques such as support vector machines (SVM), random forests, and neural networks are used to analyze large datasets and identify complex patterns in breast cancer progression. These machine learning models can integrate diverse types of data, including genetic, transcriptomic, and clinical information, to improve prediction accuracy and develop personalized treatment plans. Multiomics approaches combine data from genomics, proteomics, and transcriptomics to develop comprehensive predictive models for Stage III breast cancer^11^. These models leverage information from DNA sequencing, RNA expression profiles, and protein expression patterns to create more accurate and individualized survival predictions. Multiomics models can uncover novel biomarkers and offer a more detailed understanding of breast cancer biology, leading to improved prognostic tools and treatment strategies. Integrating predictive models into clinical practice involves translating model predictions into actionable treatment decisions. This includes using models to stratify patients based on their risk of recurrence, selecting appropriate therapies, and monitoring treatment responses. Clinical decision support systems (CDSS) that incorporate predictive models can assist healthcare providers in making informed decisions and improving patient care.

### Biomarkers for survival prediction in stage III breast cancer

Biomarkers are biological molecules or characteristics that can be measured to provide information about the presence, progression, or treatment response of cancer. In Stage III breast cancer, biomarkers are critical for predicting survival outcomes, guiding treatment decisions, and personalizing patient care^31^. They offer insights into the molecular and genetic underpinnings of the disease, which can be used to develop more effective treatment strategies and improve patient outcomes. Hormone receptor status, including estrogen receptor (ER) and progesterone receptor (PR) expression, plays a significant role in determining prognosis and guiding treatment for Stage III breast cancer patients^32^. Tumors that are ER-positive and PR-positive often respond well to hormone therapies such as tamoxifen or aromatase inhibitors, which can improve survival outcomes. Conversely, hormone receptor-negative tumors may require more aggressive treatment strategies, including chemotherapy and targeted therapies. The Human Epidermal Growth Factor Receptor 2 (HER2) is a critical biomarker in breast cancer that is associated with aggressive disease and poor prognosis^33^. HER2-positive tumors overexpress the HER2 protein, which can drive cancer progression. Targeted therapies, such as trastuzumab and pertuzumab, are effective treatments for HER2-positive stage III breast cancer and have been shown to improve survival rates and reduce the risk of disease recurrence.

The Ki-67 proliferation index measures the fraction of tumor cells actively undergoing mitosis. High Ki-67 levels are associated with more aggressive tumor behavior and poorer prognosis. In Stage III breast cancer, the Ki-67 index is used to assess tumor growth rate and predict response to treatment^34^. High Ki-67 levels can indicate a need for more intensive therapies and are often considered in conjunction with other biomarkers for comprehensive survival prediction. Oncotype DX and MammaPrint are genomic assays that evaluate the expression of a panel of genes to predict the risk of cancer recurrence and guide treatment decisions. Oncotype DX provides a recurrence score based on 21 genes, while MammaPrint assesses the expression of 70 genes to classify patients as either high or low risk for recurrence. These assays are valuable tools for personalizing treatment plans and predicting long-term survival outcomes in stage III breast cancer patients. Mutations in the BRCA1 and BRCA2 genes are well-established biomarkers for breast cancer risk^35^. Patients with these genetic mutations are at a higher risk of developing stage III breast cancer and may require more aggressive treatment approaches. Genetic testing for BRCA mutations helps identify individuals who may benefit from preventative measures and targeted therapies, and is a key factor in personalized treatment strategies. Programmed Death-Ligand 1 (PD-L1) expression is a biomarker for assessing the potential efficacy of immunotherapy in breast cancer^36^. High PD-L1 expression can indicate that a tumor is responsive to immune checkpoint inhibitors, which can be used as a treatment option for stage III breast cancer. The role of PD-L1 as a biomarker in predicting treatment response and survival outcomes is an area of ongoing research. Circulating tumor cells (CTCs) and circulating tumor DNA (ctDNA) are emerging biomarkers for monitoring disease progression and predicting survival outcomes. CTCs are cancer cells that detach from the primary tumor and circulate in the blood, while ctDNA consists of fragmented tumor DNA present in the bloodstream^37^. These biomarkers provide real-time insights into disease status and can be used to predict treatment responses and recurrence risks in stage III breast cancer. Molecular subtyping divides breast cancer into distinct categories based on gene expression profiles. The Luminal A, Luminal B, HER2-enriched, and Triple-negative subtypes offer insights into tumor behavior and treatment response. Each subtype has different survival outcomes and treatment strategies, with specific biomarkers associated with each molecular profile.

### Clinical parameters of stage III breast cancer

Clinical parameters in stage III breast cancer are essential for assessing the extent of disease, determining the prognosis, and guiding treatment decisions^38^. These parameters, which are based on clinical and pathological characteristics, help healthcare professionals stratify patients and develop personalized treatment plans. The size of the primary tumor (measured in centimeters) is a fundamental clinical parameter. Larger tumors are generally associated with a poorer prognosis. Tumor size is typically determined through physical examination, mammography, ultrasound, or MRI. The involvement of regional lymph nodes in the axillary region is a critical factor in staging breast cancer. Lymph node status is determined through physical examination and confirmed by techniques like sentinel lymph node biopsy (SLNB) or axillary lymph node dissection (ALND). The presence of cancer cells in lymph nodes indicates a more advanced stage and may influence treatment decisions. Hormone receptor status refers to the expression of estrogen receptors (ER) and progesterone receptors (PR) in the breast cancer cells^39^. Tumors that are hormone receptor-positive can be treated with hormone therapy, while those that are hormone receptor-negative may require different treatment approaches.

Human Epidermal Growth Factor Receptor 2 (HER2) status is determined by testing for overexpression or amplification of the HER2 protein^40^. HER2-positive breast cancers may be treated with targeted therapies like Herceptin (trastuzumab) in addition to standard treatments. Histologic grade assesses the degree of differentiation of the cancer cells. A higher grade indicates more aggressive tumor behavior. Tumors are typically graded on a scale from 1 to 3. The clinical stage of breast cancer is determined based on the size of the tumor, lymph node involvement, and the presence of distant metastases. In stage III breast cancer, tumors are locally advanced and may have spread to nearby lymph nodes, but they have not yet metastasized to distant organs. Age can influence the choice of treatment and prognosis. Younger women may have different considerations, such as fertility preservation and long-term survivorship, that healthcare professionals need to address. Menopausal status is important in hormone receptor-positive breast cancer, as it affects the choice of endocrine therapy. Premenopausal and postmenopausal women may receive different hormonal treatments. The patient’s general health and the presence of other medical conditions can impact the selection and tolerance of cancer treatments. The Eastern Cooperative Oncology Group (ECOG) performance status assesses a patient’s ability to carry out daily activities^41^. It is important for determining the patient’s fitness for certain treatment regimens. The symptoms experienced by the patient, such as pain, skin changes, or nipple discharge, are important for diagnosing and managing the disease. In cases where neoadjuvant chemotherapy or targeted therapy is administered before surgery, the response of the tumor to these treatments is a significant clinical parameter. A complete pathological response may influence the extent of surgery required.

### Multimodal approaches to stage III breast cancer

Multimodal approaches to stage III breast cancer involve the integration of various treatment modalities and strategies to provide patients with the most comprehensive and effective care^42^. These approaches aim to address the complexities of locally advanced breast cancer, maximize the chances of a successful outcome, and improve the quality of life for individuals facing this challenging diagnosis. Neoadjuvant therapy involves administering systemic treatments, such as chemotherapy, targeted therapy, or hormone therapy, before surgery. This approach is commonly used in stage III breast cancer to shrink the tumor, control the disease, and facilitate breast-conserving surgery. Neoadjuvant therapy also provides valuable information about the tumor’s response to treatment, which can guide subsequent decisions. Surgical intervention is a crucial component of multimodal treatment for stage III breast cancer^43^. The extent of surgery can vary, ranging from breast-conserving surgery (lumpectomy) to mastectomy, depending on factors like tumor size, location, and response to neoadjuvant therapy. Axillary lymph node dissection or sentinel lymph node biopsy may be performed to assess lymph node involvement. After surgery, radiation therapy may be recommended to target any remaining cancer cells in the breast or chest wall. Radiation is an essential component of treatment, particularly for patients who undergo breast-conserving surgery or have a high-risk of local recurrence.

Chemotherapy is a systemic treatment that may be used before surgery (neoadjuvant chemotherapy) to reduce tumor size and improve surgical outcomes or after surgery (adjuvant chemotherapy) to eliminate any remaining cancer cells. The choice of chemotherapy agents and duration depends on the specific characteristics of the tumor. Hormone therapy is typically used for hormone receptor-positive breast cancer. It may be recommended in conjunction with surgery and/or radiation therapy to block the effects of estrogen on cancer cells and reduce the risk of recurrence. In cases of HER2-positive breast cancer, targeted therapies like trastuzumab (Herceptin) are used to specifically target the HER2 protein, which can lead to more effective treatment and improved outcomes^44^. Immunotherapy is an emerging field in breast cancer treatment. Immune checkpoint inhibitors, such as pembrolizumab, are being studied in clinical trials for breast cancer and may be incorporated into treatment approaches in the future. Multimodal approaches include comprehensive supportive care to address side effects of treatment, manage symptoms, and improve the overall quality of life for patients. This may include psychosocial support, pain management, and symptom control. Participation in clinical trials may be offered to eligible patients as part of a multimodal approach. These trials explore new treatment strategies, targeted therapies, and immunotherapies to further advance the care of stage III breast cancer patients. Regular follow-up appointments, imaging, and laboratory tests are crucial to monitor the patient’s response to treatment and to detect any potential recurrence or metastasis at an early stage.

### Challenges

One of the primary challenges in predicting survival outcomes in stage III breast cancer is the lack of standardized data collection and integration across healthcare systems^45^. The use of various electronic health record systems and disparate databases can hinder the seamless sharing of clinical, genetic, and imaging data. Developing standardized data formats and implementing interoperable systems are essential to harness the full potential of predictive tools. Breast cancer is a genetically heterogeneous disease with various subtypes and molecular characteristics. To improve prediction accuracy, genetic markers and gene expression profiles need to be better refined and stratified based on specific subtypes. There are disparities in access to advanced genetic testing and imaging techniques, both within and between different regions and populations. Ensuring equitable access to these tools is essential for providing personalized care and making survival outcome predictions universally relevant. Some genetic tests and advanced imaging modalities can be expensive, potentially limiting their accessibility. Cost-effectiveness studies and healthcare policy decisions need to be aligned to ensure that these tools are used judiciously and cost-efficiently. Predicting survival outcomes can be complicated by the dynamic nature of cancer progression^46^. Tumors can evolve, develop resistance to treatments, and undergo genomic changes. Long-term follow-up and the development of tools that can adapt to these changes are necessary for accurate predictions. With the increasing reliance on genetic and clinical data, privacy and security issues become more prominent. Ensuring the protection of patients’ sensitive data while promoting data sharing and research collaboration is a delicate balance.

### Future directions

Advances in precision medicine are driving the development of tailored therapies based on genetic markers and molecular profiling^47^. Future directions include the identification of novel biomarkers, the expansion of targeted therapies, and the integration of immunotherapies to further personalize treatment plans for stage III breast cancer patients. Artificial intelligence (AI) and machine learning algorithms are playing an increasingly significant role in predicting survival outcomes. These technologies can analyze vast datasets, discover new associations, and refine predictive models. Future directions include the integration of AI-based decision support tools into clinical practice to enhance prediction accuracy. Liquid biopsies, which involve the analysis of circulating tumor DNA and other biomarkers in blood samples, hold promise for real-time monitoring and early detection of disease recurrence. Future directions include the development of more sensitive and specific liquid biopsy techniques for stage III breast cancer^48^. Radiomics, which extracts quantitative features from medical images, continues to evolve. Future directions include the refinement of radiomic models to better predict treatment response and survival outcomes, potentially reducing the need for invasive biopsies. The integration of genetic, imaging, and clinical parameters will become increasingly sophisticated. Future directions include the development of comprehensive models that combine these elements to provide a holistic assessment of each patient’s condition and predict survival outcomes with higher precision. Gathering and analyzing longitudinal data, including patient outcomes over extended periods, will provide a more nuanced understanding of stage III breast cancer and its treatment^49^. Future research should focus on the creation of extensive databases that capture patient trajectories. International collaboration among researchers, clinicians, and healthcare systems is essential to overcome challenges related to data sharing, disparities in access, and the validation of predictive tools. Collaborative efforts will enable the development of more universally applicable predictive models.

## Conclusion

Biomarkers such as ER, PR, HER2, and Ki-67 offer significant insights into tumor biology and patient prognosis. Hormone receptor statuses (ER and PR) are integral in directing endocrine therapy, while HER2 positivity indicates the need for targeted treatments with agents like trastuzumab. The Ki-67 proliferation index provides valuable information about tumor aggressiveness, which is essential for tailoring treatment strategies. Emerging genomic assays, including Oncotype DX and MammaPrint, provide a molecular portrait of the tumor, helping predict recurrence risks and guiding adjuvant therapy decisions. These biomarkers not only enhance our ability to stratify patients but also enable personalized treatment approaches that align with individual tumor characteristics and patient needs. Predictive models are increasingly sophisticated, incorporating diverse data sources such as genetic, molecular, and clinical variables to forecast outcomes and optimize therapeutic strategies. These models leverage advanced statistical methods and machine learning techniques to predict disease progression and patient survival with high accuracy. The integration of these predictive tools into clinical practice holds the promise of more precise, individualized treatment plans that improve overall patient outcomes.

## Data Availability

Not applicable.
